# Effective control and probe of Néel order in polycrystalline NiO films: a combined approach to study antiferromagnets

**DOI:** 10.1038/s41598-026-37152-3

**Published:** 2026-01-23

**Authors:** Chun-Chieh Hsu, Yu-Chen Lin, I-Yu Cheng, Shuan-Cheng Mai, Danru Qu, Alexander J. Grutter, Margaret Kane, Yuri Suzuki, Yu-Lon Lin, Chao-Yao Yang

**Affiliations:** 1 Department of Materials Science and Engineering, National Yang Ming Chiao Tung University, Hsinchu, 300093 Taiwan; 2 Department of Physics, National Taiwan University, Taipei, 10617 Taiwan; 3 Center for Condensed Matter Sciences, National Taiwan University, Taipei, 10617 Taiwan; 4 Center of Atomic Initiatives for New Materials, National Taiwan University, Taipei, 10617 Taiwan; 5 NIST Center for Neutron Research, National Institute of Standards and Technology, Gaithersburg, MD 20899 USA; 6 Geballe Laboratory for Advanced Materials, Stanford University, Stanford, CA 94305 USA; 7 Department of Applied Physics, Stanford University, Stanford, CA 94305 USA; 8 Center for Emergent Functional Matter Science, National Yang Ming Chiao Tung University, Hsinchu, 300093 Taiwan

**Keywords:** Materials science, Nanoscience and technology, Physics

## Abstract

Antiferromagnetic (AFM) materials are promising for next-generation spintronic applications, however, practical implementation remains challenging due to difficulties in controlling the Néel order and the lack of sensitive, device-compatible readout techniques. In this work, we demonstrate spin Hall magnetoresistance (SHMR) as an effective and versatile approach for probing Néel order, even in polycrystalline AFM films. Using NiO/Pt and LaNiO_3_/Pt bilayers as model systems, we show that SHMR measurements can detect the Néel temperature (T_N_), monitor spin-flop transition, and reveal field-induced orientation of the Néel order. Crucially, we establish that the control of Néel order with non-volatility is effective when initiated from the “soft” AFM phase at elevated temperature (380 K) via field-cooling (FC), enabling robust alignment of Néel order without the need for adjacent heavy metals as spin current sources. This spin-current-free control greatly expands the flexibility and scalability of AFM device design, especially in the form of polycrystalline microstructure. The results highlight the combined FC and SHMR technique as a powerful and flexible platform for both manipulation and sensitive readout of AFM states, providing a reliable basis for the design and characterization of a broad class of AFM materials and devices.

**Supplementary Information:**

The online version contains supplementary material available at 10.1038/s41598-026-37152-3.

## Introduction

Antiferromagnetic (AFM) materials have attracted considerable attention for next-generation spintronic applications due to their intrinsic advantages, including ultrafast spin dynamics, robustness against external magnetic fields, and the absence of stray fields^1,2^. These properties make AFMs ideal candidates for high-speed, densely integrated spintronic devices with minimal cross-talk. However, practical implementation remains challenging primarily due to the difficulty in controlling and detecting their internal spin structures, particularly the Néel order, which is inherently invisible to conventional magnetometry owing to the nature of zero net magnetization. Unlike ferromagnets (FMs), AFMs require indirect and often complex techniques for manipulation and readout of their spin configurations^3–6^. Recent strategies to address this issue include the use of spin–orbit torques (SOT)^7,8^, exchange-spring coupling^9–11^, and strain engineering^12,13^, which have shown promise in epitaxial or single-crystalline systems. Nonetheless, these methods often depend on well-defined crystallographic orientations or intricate multilayer architectures, limiting their compatibility with scalable, real-world devices. In contrast, polycrystalline AFMs offer better integration with large-area deposition techniques but introduce new challenges in deterministic control due to random grain orientations and competing magnetic anisotropies.

This work demonstrates a robust and scalable approach to shape and probe the Néel order in polycrystalline NiO films by combining a field-cooling (FC) process with spin Hall magnetoresistance (SHMR). SHMR arises from the interaction between spin currents in a heavy metal and the spin order of an adjacent magnetic layer^14,15^, enabling axis-sensitive and device-scale detection of magnetic configurations. By applying a magnetic field during cooling from the paramagnetic phase, the re-orientation of the Néel order transverse to the field direction was observed.^1,9,14,16^ As the system cools below the Néel temperature (Tₙ), a clear SHMR signal emerges with its magnitude strongly dependent on the NiO thickness and field strength. This allows precise probing of the Néel order and its thermal evolution. To further validate the generality of this method, we applied the SHMR-FC approach to a (111)-oriented LaNiO_3_/Pt bilayer grown on LaAlO_3_ substrate^17^, in which the AFM ordering remains less explored. A distinct SHMR signal was observed below ~ 100 K, indicating the onset of magnetic ordering and confirming the broader applicability of this SHMR-FC technique. Collectively, our results demonstrate SHMR combined with FC as a versatile platform for detecting, tuning, and understanding AFM spin configurations in technologically relevant material systems.

## Results

**Fig. 1:**
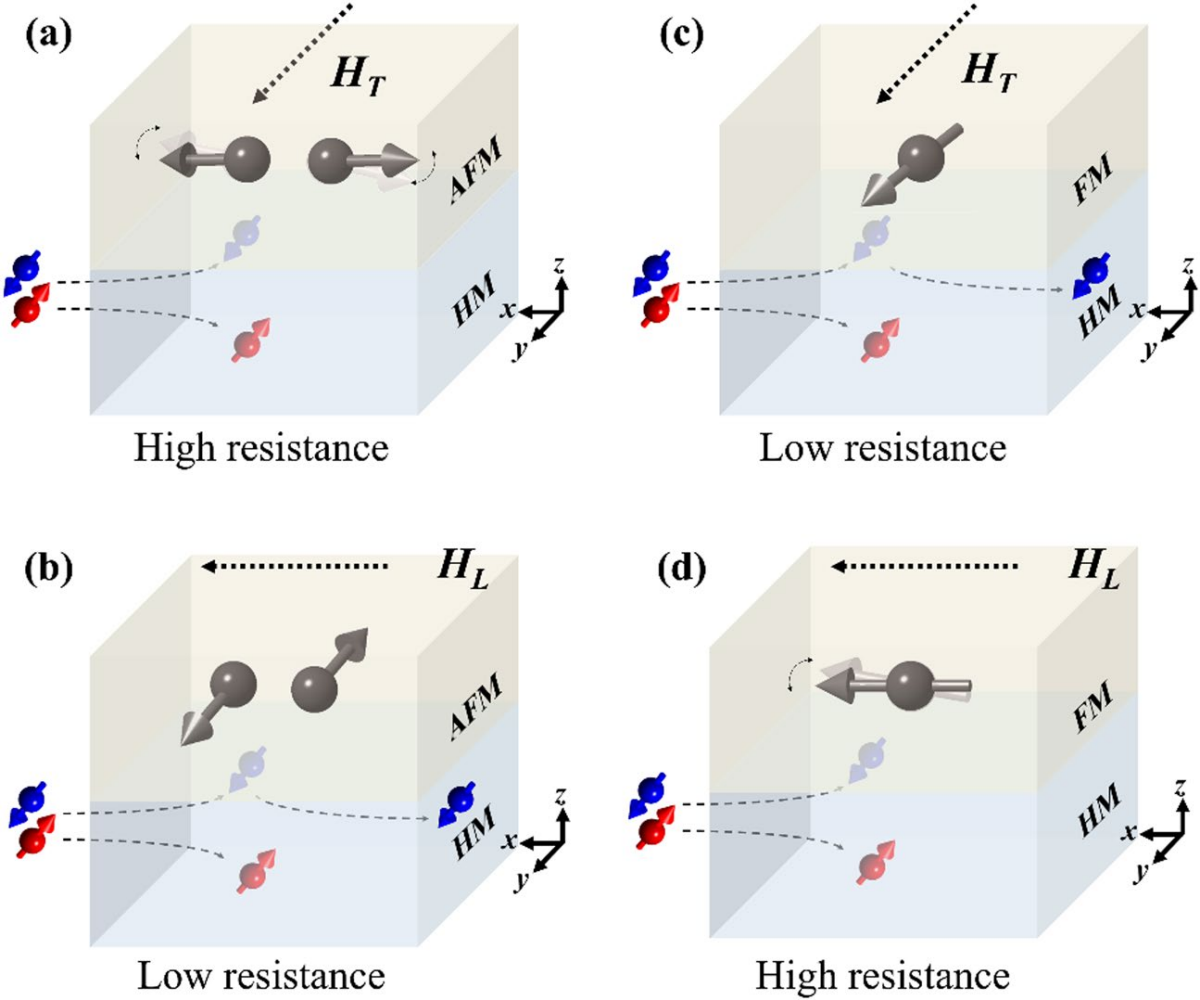
Schematic illustration of the SHMR effect in HM/AFM and HM/FM bilayers. In the HM/AFM bilayer, the Néel order is aligned (**a**) parallel and (**b**) transverse to the applied current. The orthogonal spin–Néel order correlation in (**a**) induces spin absorption via spin torque transfer, perturbing the AFM moments and resulting in a high-resistance state. In contrast, the collinear spin–Néel order correlation in (**b**) leads to spin reflection and inverse spin Hall effect (ISHE) charge current generation in the HM, yielding a low-resistance state. These configurations arise from the AFM spin-flop dynamics and are controlled by applying magnetic fields along the y- and x-directions, denoted as *H*_*T*_ and *H*_*L*_​, respectively. In the HM/FM bilayer, the FM magnetization aligns (**c**) transverse and (**d**) parallel to the applied current, leading to low- and high-resistance states, respectively, due to the reversed spin absorption/reflection behavior compared to the HM/AFM system.

Figure 1a and b schematically illustrate the concept of SHMR in a heavy-metal (HM)/AFM bilayer, where the Néel order is aligned parallel or transverse to the applied current, respectively. These configurations are established by applying in-plane magnetic fields *H*_*T*_​ and *H*_*L*_, and follow the conventional spin-flop mechanism observed in AFMs^1,9,14,16^. In particular, the sputtered NiO film exhibiting easy-plane anisotropy favors a Néel orientation perpendicular to the field to minimize Zeeman energy through spin canting (see Supporting Information S1)^14,16,18^. According to the SHMR mechanism, the resistance states *R*_*T*_ and *R*_*L*_​ (corresponding to resistance states taken in a transverse *H*_*T*_ and longitudinal *H*_*L*_ field, respectively) are expected to diverge, where the *R*_*T*_ is higher than *R*_*L*_​​. This is due to spin absorption at the AFM/HM interface, which is more pronounced when the spin polarization is perpendicular to the Néel order, leading to spin dephasing and higher resistance (Fig. 1a). In contrast, when the spin polarization aligns with the Néel order (Fig. 1b), spin absorption is suppressed and results in enhanced spin reflection. The reflected spins generate additional charge current via the inverse spin Hall effect, hence reducing the overall resistance of the device^14,15^. Thus, the ratio between spin absorption and reflection at the interface governed by relative spin–Néel order correlation serves as the origin of SHMR, enabling an axis-sensitive probing of AFM order. Notably, if the AFM is replaced with an FM layer, the SHMR polarity will be reversed (Fig. 1c and d) since FM magnetization prefers to align directly with the applied field, reversing the spin absorption/reflection symmetry relative to the AFM/HM bilayer. This reversal arises because the FM magnetization energetically favors alignment parallel to the applied magnetic field, in contrast to the AFM case where the Néel order aligns transverse to the field due to spin-flop dynamics. As a result, the symmetry of spin absorption and reflection at the interface is reversed between AFM/HM and FM/HM bilayers, leading to opposite SHMR signs.

**Fig. 2:**
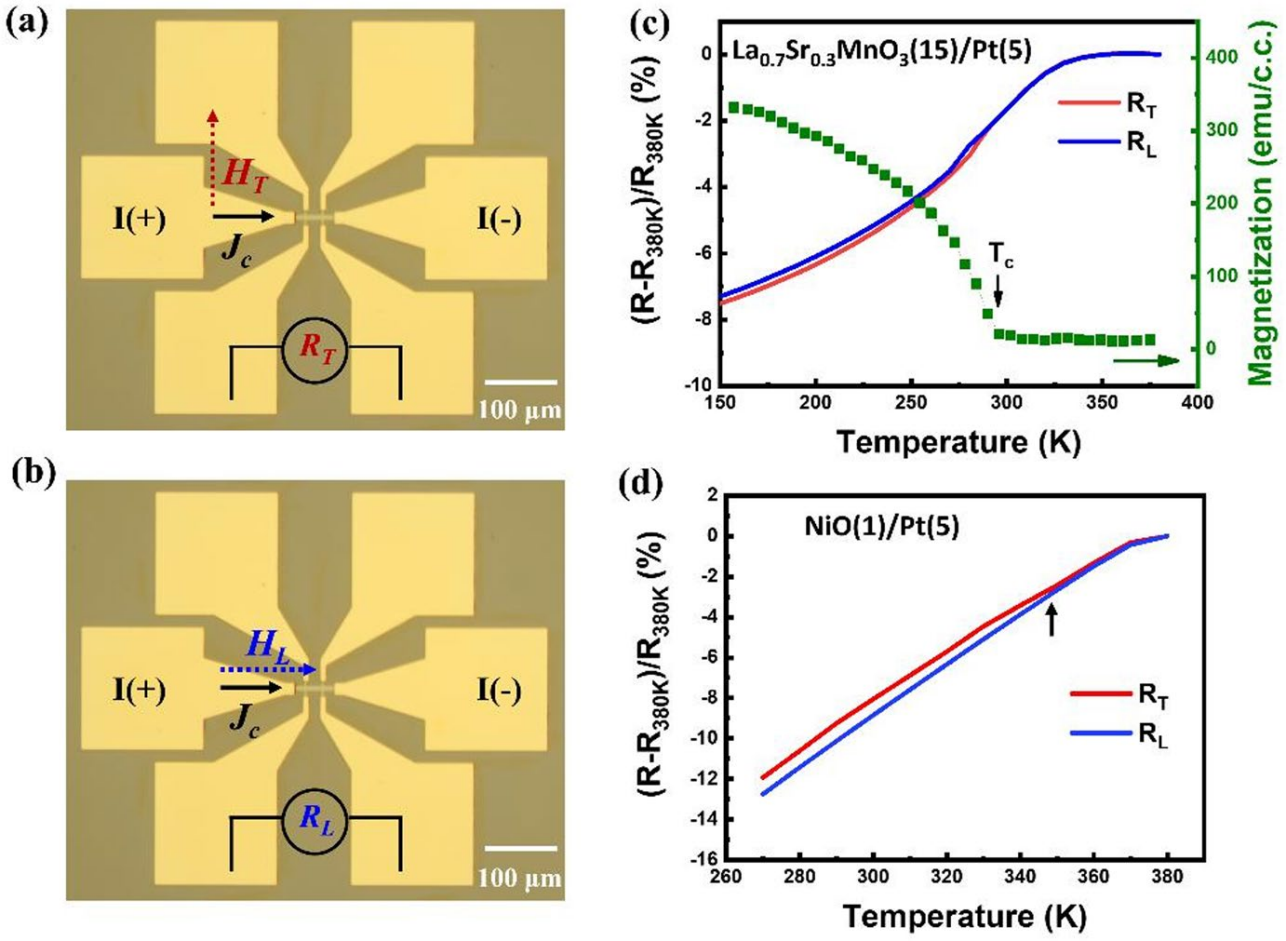
Optical microscope images of Hall bar devices used for SHMR measurements under (**a**) transverse and (**b**) longitudinal field-current geometries. The probe current was fixed at 1 mA and the applied magnetic field at 3 T for SHMR characterization. The resistance measured under configurations (**a**) and (**b**) are denoted as *R*_*T*_ and *R*_*L*_​, respectively. (**c**) Temperature-dependent resistance states (*R*_*T*_ and *R*_*L*_​) of an LSMO/Pt bilayer (15 nm/5 nm) obtained using the experimental geometries in (**a**) and (**b**) with FC, alongside the magnetization versus temperature curve. (**d**) Temperature-dependent resistance states of a NiO/Pt bilayer (1 nm/5 nm) measured with FC at 3 T from 380 K, following the configurations in (**a**) and (**b**).

The experimental geometry of SHMR is shown in Fig. 2a and b, where optical microscope images depict two orthogonal device configurations used to collect *R*_*T*_ and *R*_*L*_​, corresponding to transverse and longitudinal alignment of the applied magnetic field (*H*) with respect to the probe current (*J*_*c*_​). Measurements were performed on two identical devices under the same thermal and magnetic conditions, but in an orthogonal geometry. To validate the opposite SHMR effects predicted for AFM and FM-based bilayers, we begin with examining an La_0.7_Sr_0.3_MnO_3_ (LSMO)/Pt bilayer as a model system for FM/HM bilayer. Figure 2c presents temperature-dependent *R*_*T*_/*R*_*L*_​ of the LSMO/Pt bilayer, taken with FC from 380 K under a 100 mT field alongside its temperature-dependent magnetization curve (green). Note, the resistance at 380 K (*R*_*380K*_) was used as the reference for normalization, since the LSMO is paramagnetic above its Curie temperature T_C_​ and thus exhibits negligible SHMR. Upon cooling, the magnetization sharply increases around 290 K, indicating the restored FM order of LSMO. Concurrently, a clear divergence between *R*_*T*_ and *R*_*L*_​​ emerges, consistent with the SHMR correlation expected from Fig. 1c (low-resistance) and Fig. 1d (high-resistance). The result confirms the SHMR originating from the FM ordering in LSMO/Pt bilayer. Having established the validity of SHMR in an FM system, we applied the same FC-SHMR technique to probe the AFM ordering in NiO/Pt bilayers. As shown in Fig. 2d, SHMR measurements performed with FC from 380 K under a 3 T magnetic field reveal a distinct divergence between *R*_*T*_ and *R*_*L*_​​ ​at around 350 K. This may be associated with the Néel temperature (T_N_​) of a 1 nm-thick NiO film, denoted as NiO(1), as revealed by the previous literature^14,19,20^. Note that, SHMR is an axial probe for the Néel order, so that is independent of types of heavy-metal with opposite spin Hall angle (see Supporting Information S2). The relative evolution on *R*_*T*_ and *R*_*L*_​​ matches the expected configuration depicted in Fig. 1a and b, which is opposite to that of the FM case. The result supports the interpretation that the Néel order in NiO aligns orthogonal to the applied field due to its easy-plane anisotropy (see Supporting Information S3)^14,16,18^. Note that, due to the intrinsic sensitivity limitation of the magnetometer, temperature-dependent magnetization measurements for the NiO thin films are not capable of resolving the magnetization change as a function of temperature. Therefore, the FM LSMO sample serves as a reference system, for which both temperature-dependent magnetization and SHMR measurements are experimentally accessible. As shown in Fig. 2c, this comparison demonstrates the feasibility of the SHMR–field-cooling approach and supports the identification of the characteristic temperature in SHMR as corresponding to the magnetic ordering temperature.

**Fig. 3:**
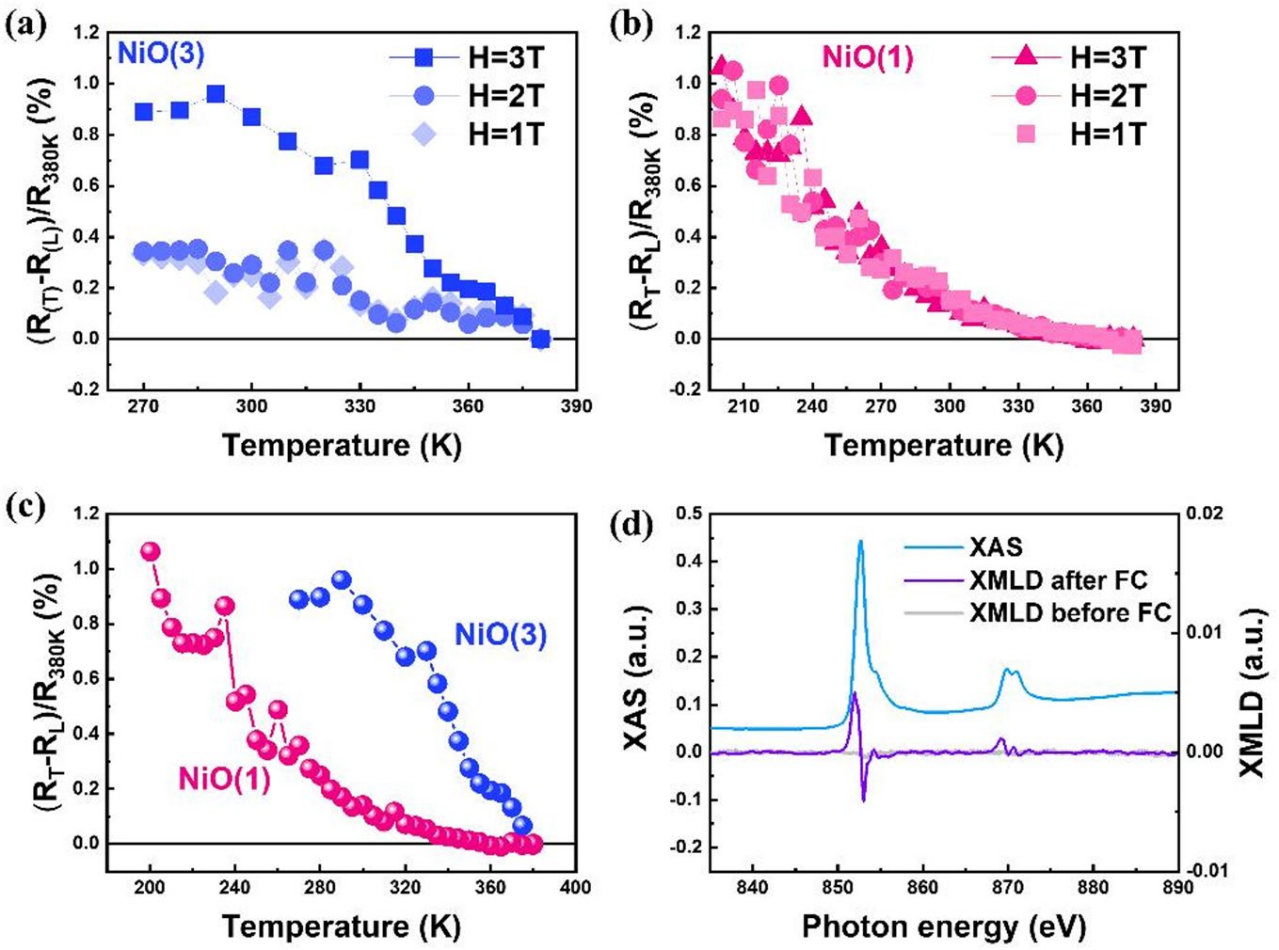
(**a**) SHMR ratio ((*R*_*T*_*−R*_*L*_)/*R*_*380 K*_)×100% of NiO(3)/Pt bilayer measured via FC under various applied magnetic fields. (**b**) SHMR ratio of NiO(1)/Pt bilayer under the same conditions. (**c**) Superimposed SHMR ratios of NiO(1)/Pt and NiO(3)/Pt bilayers measured via FC at 3 T, highlighting thickness-dependent T_N_ transitions. (**d**) X-ray absorption spectroscopy (XAS) and X-ray magnetic linear dichroism (XMLD) spectra at the Ni L_2,3_ edges collected at room temperature with and without FC treatment for NiO(3)/Pt bilayer. XMLD spectra were obtained by subtracting XAS of linear polarization parallel (*L*_*P*_) to the applied magnetic field from the one transverse (*L*_*T*_). Note: The field-dependent measurements were all performed from the low field (1 T) to high field (3 T) to avoid any magnetic irreversibility at the early stage of the experiment.

To further explore the field dependence of SHMR during FC, Fig. 3a presents the normalized SHMR ratio, defined as (*R*_*T*_−*R*_*L*_)/*R*_*380K*_​ for the NiO(3)/Pt bilayer with varying magnetic field strengths. The observation on *R*_*T*_>*R*_*L*_​ is consistent with the configurations in Fig. 1a and b. In all cases, the SHMR ratio remains positive and generally increases with field strength, suggesting that the magnetic field during FC aligns the Néel order orthogonally to the field direction through the spin-flop mechanism. Note that, the “positive” SHMR we defined thorough the manuscript is to show the result of higher *R*_*T*_ over *R*_*L*_, which is not the same as the definition obtained from the regular SHMR of FM/HM bilayer. This definition is intentionally chosen to facilitate a direct comparison of resistance states associated with different degrees of Néel-order orientations, as the absolute value, in AFM/HM systems throughout the manuscript. The opposite SHMR polarity observed between AFM/HM and FM/HM bilayers originates from their fundamentally different magnetic responses to the applied field: the field aligns the Néel order transverse to the field in AFMs, while it aligns the magnetization parallel to the field in FMs. Consequently, the SHMR polarity serves as a reliable indicator of the correlation between magnetic order and field direction in both AFM/HM and FM/HM bilayer systems. The enhanced SHMR ratio at higher fields in Fig. 3a further implies more effective Néel order alignment with the increasing field strength. In contrast, Fig. 3b shows the SHMR ratios of the NiO(1)/Pt bilayer under similar FC conditions. Unlike the NiO(3) case, the SHMR signal displays negligible dependence on field strength. Figure 3c shows the superimposed SHMR curves for both bilayers taken using 3 Tesla for comparison. A clear difference is observed in the onset temperature of SHMR: ~380 K for the NiO(3)/Pt bilayer and ~ 330 K for the NiO(1)/Pt bilayer. This shift in transition temperature confirms a thickness-dependent Tₙ for NiO thin film, coinciding with the known reduction in AFM order robustness in ultrathin NiO films^14,18–20^. These observations indicate that FC from above Tₙ is essential for effective “shaping” of the Néel order. When the FC temperature exceeds Tₙ, i.e. 380 K for NiO(1), even relatively low fields can function as a trigger to successfully guide the formation of transverse Néel order alignment, resulting in a saturated SHMR response across all investigated field strengths. In contrast, for NiO(3), 380 K may lie just below its actual Tₙ, therefore, the alignment of Néel order becomes more gradual and sensitive to field strength due to the presence of varied distribution of local AFM anisotropy among grains^21,22^. This suggests the observed SHMR response may arise from the field-assisted AFM domain repopulation and partial Néel order reorientation, rather than a coherent spin-flop transition in the entire NiO(3) film.

To directly confirm the field-induced Néel order reorientation, Fig. 3d presents X-ray magnetic linear dichroism (XMLD) spectra at the Ni L_2_,_3_ edges of the NiO(3)/Pt bilayer before and after FC under a 3 T field. As anticipated, none of distinguishable XMLD signal is observed in the as-grown sample (grey curve). However, a characteristic XMLD spectrum (purple curve) obtained from the difference between X-ray absorption spectra measured with linear polarization parallel (*L*_*P*_) and transverse (*L*_*T*_) to the applied field (*L*_*P*_ –*L*_*T*_) emerges after FC. The distinct peak-to-dip feature is a signature of AFM order aligned orthogonal to the field^16,19,22^, in agreement with the SHMR results very well. Together, these findings confirm that SHMR measurements, particularly under FC, provide a powerful platform to identify Tₙ, characterize Néel order alignment, and probe spin-flop coupling in polycrystalline AFM films.

**Fig. 4:**
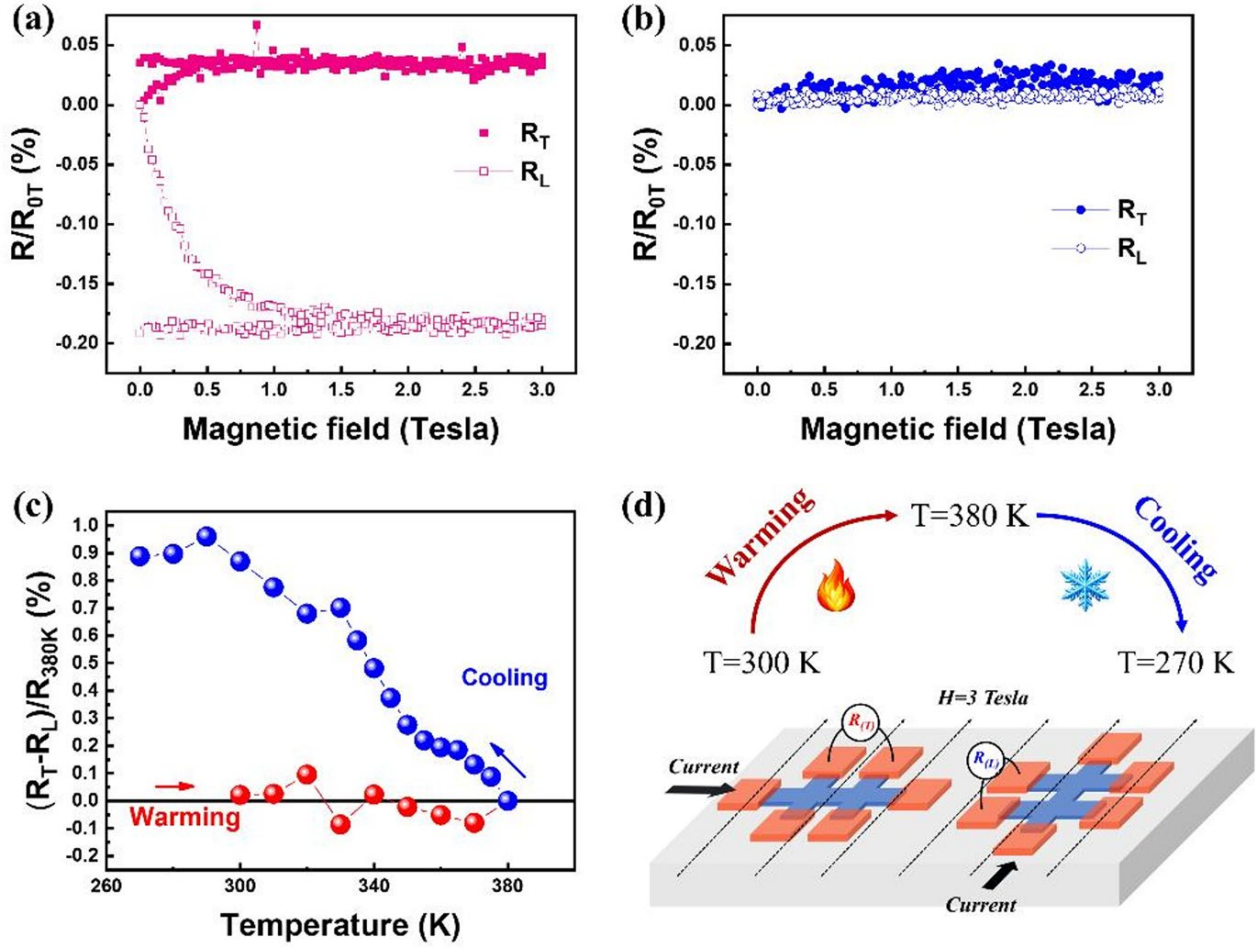
(**a**) Longitudinal resistance *R*_*L*_ and transverse resistance *R*_*T*_ of NiO(1)/Pt bilayer as a function of applied magnetic field, measured during field sweep from 0 T to 3 T and back to 0 T. (**b**) Corresponding *R*_*L*_ and *R*_*T*_ ​ for NiO(3)/Pt bilayer under the same measurement sequence. (**c**) Comparison of SHMR ratio ((*R*_*T*_*−R*_*L*_)/*R*_*380 K*_)×100% ​​of NiO(3)/Pt bilayer acquired during field warming (red) and field cooling (blue) under 3 T, illustrating enhanced Néel order alignment with field cooling. (**d**) Schematic illustration of the experimental geometry and measurement workflow for SHMR under a constant magnetic field of 3 T. The measurement sequence starts with field-warming from 300 K to 380 K, followed immediately by field-cooling from 380 K down to 270 K, while continuously recording the longitudinal and transverse resistance components. This protocol enables direct comparison of Néel order evolution during warming and cooling under identical magnetic-field conditions.

To evaluate the control the Néel order at room temperature (300 K), field-dependent SHMR measurements were performed on NiO/Pt bilayers with NiO of 1 nm and 3 nm, as shown in Fig. 4a and b, respectively. For NiO(1)/Pt bilayer, a clear divergence between *R*_*T*_ and *R*_*L*_​ evolution is observed with a “positive” SHMR, consistent with orthogonal field-Néel order correlation. It could be noticed that when the field returns to zero, both *R*_*T*_ and *R*_*L*_​ are still sustainable without considerable changes. It suggests the shaping of the Néel order is non-volatile and would be attributed to the easy-plane anisotropy of NiO film (see Supporting Information S3), which can be also supported by the XMLD result in Fig. 3d. Besides, the ideal remanence on the *R*_*T*_ and *R*_*L*_​ may also suggest the magnetostriction effect in the poly-crystalline NiO could be ignorable, otherwise both *R*_*T*_ and *R*_*L*_​ should return subsequently as a result of magnetostriction effect^23,24^. More detailed discussion on the asymmetric evolution of *R*_*T*_ and *R*_*L*_ can be found in Supporting Information S4. In contrast, the NiO(3)/Pt bilayer shows no distinguishable SHMR signal under similar conditions, suggesting the Néel order in the thicker film might be too robust to be reoriented at room temperature without thermal assistance. To compare the efficiency of Néel order alignment at different temperatures, SHMR measurements were performed with both field-warming and FC manners, as shown in Fig. 4c. The experimental flow (Fig. 4d) is as follows: A magnetic field of 3 T was firstly applied at room temperature and the sample was then heated continuously from room temperature up to 380 K under the constant field, while the SHMR was recorded as a function of temperature during the warming process. Immediately following the field-warming measurement, the magnetic field of 3 T was maintained, and the sample was cooled from 380 K down to 270 K. The SHMR was continuously collected during this cooling process. In this measurement scheme, the resistance states at 380 K serve as a reference point. This approach allows direct comparison between field-warming and field-cooling behaviors under identical magnetic field conditions and ensures consistent tracking of the Néel order evolution as a function of temperature. Consequently, a significant SHMR signal is observed with FC treatment compared to that with field-warming, indicating that the Néel order is more effectively aligned when shaped during its formation from the magnetically soft state, which adopts the similar concept as the previous work demonstrated by FeRh^25,26^ and current-induced magnetic phase transition for Néel order re-orientation^26^. This result highlights the importance of temperature in enabling controllable Néel order alignment in AFMs and underscores the utility of FC as a practical method for manipulating AFM order in spintronic devices. It should be emphasized that, because 380 K may not coincide with the Néel temperature of the NiO(3) film, the observed SHMR response is likely governed by field-assisted AFM domain repopulation and partial Néel order reorientation, rather than a coherent spin-flop transition of the entire film.

So far, it has been demonstrated that SHMR measurements combined with FC treatment provide an effective means to characterize Néel order transitions in polycrystalline NiO films, determine Tₙ, and probe spin-flop dynamics. An unsolved issue is whether this approach can be extended to other AFM oxide systems. To examine the feasibility, we performed SHMR-FC in an epitaxially grown LaNiO_3_ film on an (111)-oriented LaAlO_3_ substrate, a material system with intriguing magnetic characteristics. LaNiO_3_ is the only member of the perovskite rare-earth nickelate family that remains metallic and paramagnetic across all temperatures in bulk^27^. However, current literature suggested that LaNiO_3_ lies near a magnetic instability, particularly under epitaxial strain^28,29^. Recent studies on ultrathin (111)-oriented LaNiO_3_ films grown on LaAlO_3_ substrates have reported emergent magnetic features, including a hysteretic anomalous Hall effect, which may be indicative of weak AFM order or a vanishingly small net magnetization^28,29^. Yet, the ultrathin nature of these films renders conventional magnetic probes such as neutron diffraction ineffective for definitive characterization.

**Fig. 5:**
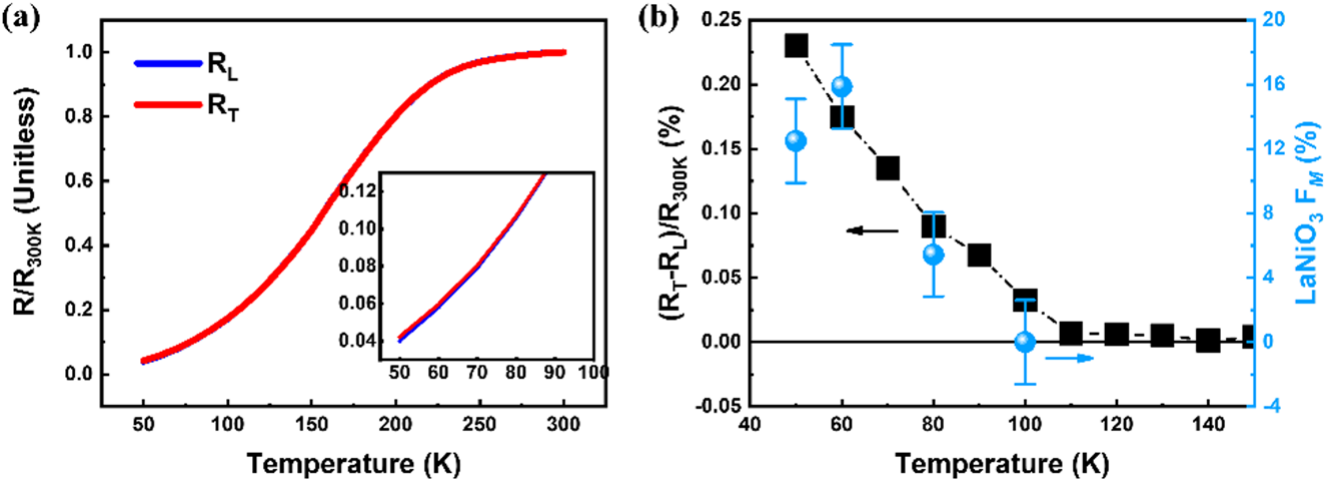
(**a**) Resistance states *R*_*L*_ and *R*_*T*_​ and (**b**) corresponding SHMR ratio ((*R*_*T*_*−R*_*L*_)/*R*_*380 K*_)×100%​​ measured with FC under a 3 T magnetic field for the (111)-oriented LaNiO_3_ (6)/Pt bilayer. The cooling started from 300 K, with the resistance difference between *R*_*L*_ and *R*_*T*_​ emerging below ~ 100 K, as highlighted in the inset of (**a**). Blue dots represent magnetic signals measured by low-energy muon spin relaxation (*F*_*M*_) [Ref.^17^], overlaid for comparison with the SHMR data.

To examine the sensitivity of SHMR in detecting subtle magnetic order in LaNiO_3_, FC-SHMR measurements were performed on the LaNiO_3_(6 nm)/Pt bilayer under a 3 T magnetic field. Figure 5a and b present the corresponding resistance traces and the derived SHMR ratio. A non-zero SHMR signal emerges near 100 K and increases with further cooling, displaying the polarity consistent with the AFM/HM bilayer geometry. This onset temperature is notably higher than previously reported values (∼50 K) from hysteretic magneto-transport and Hall effect studies^28,29^. Interestingly, our previous work using low-energy muon spin relaxation measurements also revealed a weak but statistically significant temperature dependence of the magnetically ordered volume fraction between 50 K and 100 K^17^. While this variation was not originally taken as conclusive evidence of AFM order above 50 K, the emergence of SHMR below 100 K with the positive polarity suggests the presence of AFM-like correlations in a minority phase following the spin-flop mechanism that haven’t been captured by other methods before. The findings highlight the high sensitivity of SHMR to even marginal AFM volume fractions, making it an attractive tool for detecting elusive magnetic order in ultrathin films. The applicability of SHMR combined with FC in LaNiO_3_, a system previously considered magnetically inactive, suggests that this methodology can be generalized to a broad range of studying AFM materials.

## Discussion

This work demonstrates that FC can serve as an effective strategy for collectively shaping the Néel order in polycrystalline NiO films, thereby extending the applicability of FC beyond conventional FM systems and epitaxially grown AFMs. While FC is routinely employed in FMs to study phenomena such as superparamagnetism and spin-glass behavior^30,31^, its effectiveness in AFMs, especially in polycrystalline films, is not comprehensively studied. In such systems, the random distribution of local magnetocrystalline anisotropy axes may strongly pin the Néel vector within individual grains, potentially suppressing macroscopic alignment, as schematically depicted in Supplementary Information 3.

In the present case, the observed SHMR response indicates that FC can nonetheless induce a collective Néel order reorientation in NiO, likely enabled by a relatively weak effective anisotropy and thermally softened AFM order near the Néel temperature. The degree of Néel order alignment depends on both the applied field strength and the overall AFM robustness, which can be tuned via film thickness. These results highlight that FC in polycrystalline AFMs is conditional rather than universal, but can be highly effective under appropriate material and thermal conditions.

It is also important to distinguish the roles of NiO and LaNiO_3_ in this study. The sputtered NiO films provide a direct demonstration of the applicability of the SHMR–FC approach to polycrystalline antiferromagnets, where broad distributions of local anisotropy and domain structures are expected. In contrast, the LaNiO_3_/Pt bilayers investigated here consist of (111)-oriented epitaxial thin films stabilized under specific strain conditions. Note that, the LaNiO_3_ results therefore serve primarily as a demonstration of sensitivity of the SHMR–FC approach, demonstrating its capability to detect the Néel temperature and spin-flop–related behavior in an epitaxial AFM system, rather than establishing applicability to polycrystalline LaNiO_3_ films.

In addition, for some spintronics applications, control over the Néel order orientation is essential for optimizing spin transport and enhancing spin-orbit torque (SOT) efficiency in magnetic heterostructures^16^. In our previous study, it shows the Néel order in NiO could be reoriented via the SOT generated from the bottom W electrode in a W/NiO/CoFeB trilayer^16^. This spin-current-driven manipulation on NiO Néel order enabled enhanced spin transmission through magnon dynamics in the NiO, which in turn significantly amplified the SOT applied to the adjacent CoFeB^16^. However, this approach inherently relied on the presence of a spin-current source (W) to induce reorientation of the Néel order in NiO adjacent, thereby limiting design flexibility and scalability in multilayer device architectures. On the contrary, the FC technique presented in this work provides a spin-current-free route to deterministically align the Néel order. By cooling from above the T_N_ under a magnetic field, AFM domains undergo spin-flop reorientation, yielding long-range magnetic order that persists at room temperature. This enables programmable control over the Néel order independent of spin injection, opening a new avenue for the integration of AFMs into memory, logic, and energy-harvesting devices with simplified architecture. Moreover, SHMR proves to be a powerful, sensitive, and device-compatible probe for characterizing AFM materials. It enables indirect detection of spin orientation and transitions, offering clear insights into the spin-flop mechanism and AFM robustness even in systems where conventional magnetic probes fail such as ultrathin or polycrystalline films. The applicability of SHMR to both well-studied (NiO) and emerging (LaNiO_3_) AFM systems underscores its versatility. Together, the SHMR-FC combination represents a robust platform for controlling and probing AFM spin textures. This dual-functionality approach not only facilitates fundamental studies of AFM spin dynamics but also serves as a practical toolset for developing next-generation spintronic technologies based on AFMs.

## Summary

In this work, we demonstrate SHMR combined with FC as an effective and versatile approach for both controlling and probing the Néel order in AFM thin films. Through systematic investigations of NiO/Pt and LaNiO_3_/Pt bilayers, we demonstrate that SHMR enables sensitive detection of the Tₙ, clear observation of spin-flop dynamics, and determination of the AFM spin orientation relative to external magnetic fields. The results confirm that shaping the Néel order is most effective when performed from the high-temperature “soft” AFM phase via FC, particularly in polycrystalline NiO. Importantly, the FC approach enables deterministic control of the AFM spin configuration without requiring a spin current source, providing a flexible and energy-efficient alternative to SOT-based manipulation schemes. Altogether, this study positions the SHMR-FC methodology as a powerful and broadly applicable platform for advancing AFM-based spintronic technologies. It offers a unified route to both manipulate and characterize AFM order, thereby facilitating the development of scalable, next-generation spintronic devices.

## Materials and methods

### Materials and device fabrication

 Film stacks with the configuration of Si/SiO_2_/Ta(2)/NiO(*t*)/Pt(5), where *t* = 1, 2, and 3 nm, were deposited via magnetron sputtering under a base pressure of 1 × 10^− 8^ Torr. Thicknesses (in nanometers) are indicated in parentheses. The Ta and Pt layers were deposited using DC sputtering at 30 W in a 5 mTorr Ar atmosphere, serving as the adhesion and spin current source layers, respectively. The NiO layers were deposited via RF sputtering at 150 W. During deposition, substrates were continuously rotated at 10 rpm to ensure uniform film growth. For comparison studies, 15 nm La_0.7_Sr_0.3_MnO_3_ (LSMO) and 6 nm LaNiO_3_ (LNO) films were grown using pulsed laser deposition (PLD) on (001)-oriented SrTiO_3_ and (111)-oriented LaAlO_3_ substrates, respectively. The (111)-oriented LNO films were deposited at 780 °C under an oxygen partial pressure of 20 mTorr using a 248 nm KrF excimer laser (fluence: 0.8 J/cm²). Subsequently, a 5 nm Pt layer was deposited by DC sputtering (30 W, 5 mTorr Ar) to complete the stack for SHMR measurements. All films were patterned into Hall bar geometries (120 μm × 10 μm) using Ar ion beam etching. Electrical contacts were fabricated by electron-beam evaporation of Ti(5)/Au(100), followed by standard photolithography and lift-off processes.

### Spin hall magnetoresistance (SHMR) measurements

SHMR measurements were performed using a Quantum Design VersaLab Physical Properties Measurement System (PPMS), equipped with a 3 Tesla superconducting electromagnet and an electrical transport module. FC treatments were carried out by applying a magnetic field either longitudinally or transversely to the current path during cooling from elevated temperatures. After FC, a constant probe current of 1 mA was applied, and the longitudinal (*R*_*L*_) and transverse (*R*_*T*_) resistances were measured using a Keithley 2000 electrometer. The SHMR ratio was defined as (*R*_*T*_–*R*_*L*_)/*R*_*380K*_, where *R*_*380K*_ is the reference resistance measured at 380 K for normalization.

### X-ray magnetic linear dichroism (XMLD)

XMLD measurements were conducted at the Ni L_2_,_3_ absorption edges using Beamline 45A1 of the Taiwan Photon Source (TPS) at the National Synchrotron Radiation Research Center (NSRRC), Taiwan. X-ray absorption spectra (XAS) were collected in total electron yield (TEY) mode with linearly polarized X-rays aligned either parallel (*L*_*P*_) or transverse (*L*_*T*_) to the current direction of the Hall bar device. The XMLD signal was extracted as the difference between the two spectra (*L*_*T*_–*L*_*P*_). The X-ray beam was focused to 1 μm × 1 μm (FWHM), enabling high-resolution spatial detection of local magnetic anisotropy, particularly at the intersection region modified by FC treatment.

## Supplementary Information

Below is the link to the electronic supplementary material.

Supplementary Material 1

## Data Availability

The authors declare that the main data supporting the findings of this study are available within the article. Extra data are available from the corresponding author upon reasonable request.
