# Supplementary material for: Effective control and probe of Néel order in polycrystalline NiO films: a combined approach to study antiferromagnets
Source: Sci Rep. 2026 Jan 23;16:6079. doi: 10.1038/s41598-026-37152-3 (PMC12901245; doi:10.1038/s41598-026-37152-3)
Supplement: Supplementary file 1 — Supplementary Material 1 [file 41598_2026_37152_MOESM1_ESM.docx]

**(Supporting Information)**

**Effective control and probe of Néel order in polycrystalline NiO films: A combined approach to study antiferromagnets**

Chun-Chieh Hsu^1^, Yu-Chen-Lin^1^, I-Yu Cheng^1^, Shuan-Cheng Mai^2,3^, Danru Qu^3,4^, Alexander J. Grutter^5^, Margaret Kane^6^, Yuri Suzuki^6,7^, Yu-Lon Lin^1^, and Chao-Yao Yang^1,8^*

^1^Department of Materials Science and Engineering, National Yang Ming Chiao Tung University, Hsinchu, 300093, Taiwan.

^2^Department of Physics, National Taiwan University, Taipei 10617, Taiwan

^3^Center for Condensed Matter Sciences, National Taiwan University, Taipei 10617, Taiwan.

^4^Center of Atomic Initiatives for New Materials, National Taiwan University, Taipei 10617, Taiwan

^5^NIST Center for Neutron Research, National Institute of Standards and Technology, Gaithersburg, Maryland 20899, USA.

^6^Geballe Laboratory for Advanced Materials, Stanford University, Stanford, California 94305, USA.

^7^Department of Applied Physics, Stanford University, Stanford, California 94305, USA

^8^Center for Emergent Functional Matter Science, National Yang Ming Chiao Tung University, Hsinchu 300093, Taiwan.

Corresponding Authors:

Prof. Chao-Yao Yang

E-mail: [cyyang8611@nycu.edu.tw](mailto:cyyang8611@nycu.edu.tw)

**Supporting information 1∣Spin-flop mechanism in NiO film with easy-plane anisotropy**

**
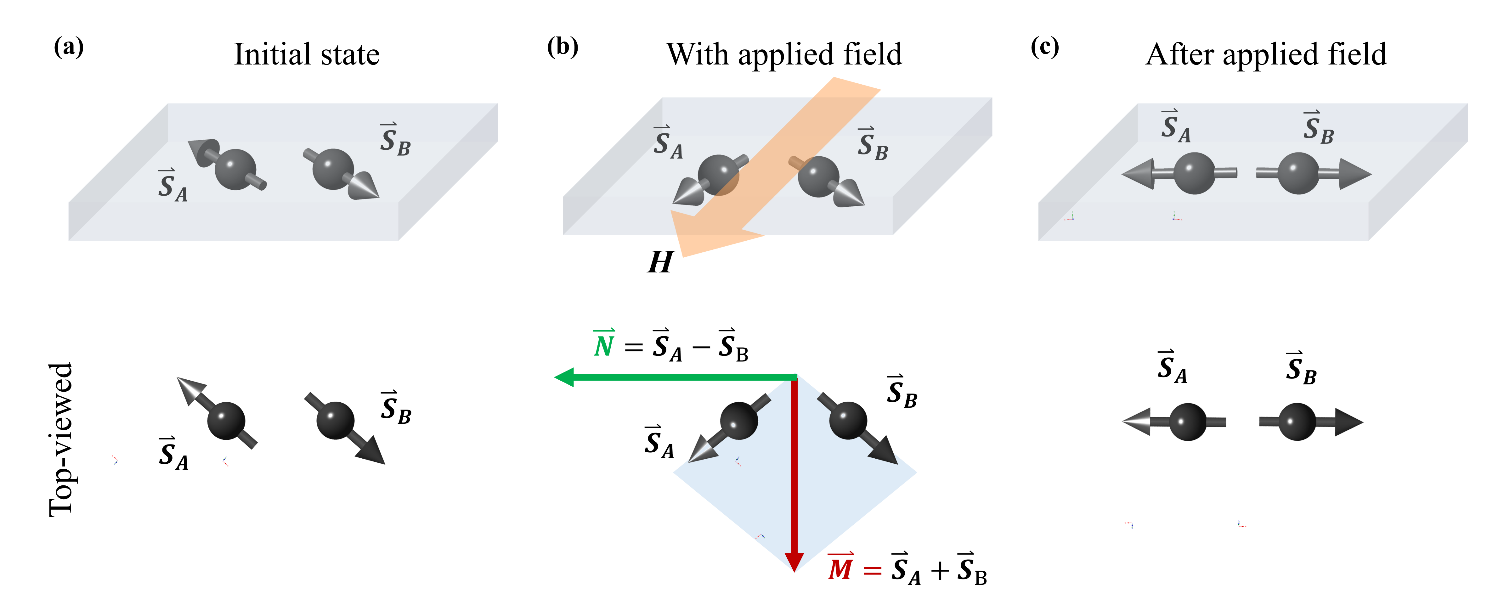
**

**Figure S1.** Schematic diagram to exhibit the spin-flop transition at (a) initial state, (b) the state during an external field applied, and (c) the state after applying field. Figures at the bottom of each panel exhibit the top-viewed image of the two-spin configurations corresponding to the different stages.

Néel order of NiO should randomly orient in the film plane in the as-deposited state because of the absence of anisotropy in the poly-crystalline film structure. In this state, local Néel order is determined by the local anisotropy in a NiO grain. **Figure S1(a)** shows a pair of AFM sub-lattice spins, denoted as $S_{A}$ and $S_{B}$, in the film plane, which are aligned deviating from the x- and y-axis at the initial state. Note, the pair of AFM sub-lattice spins can point randomly in the plane. Upon applying a field in the film plane, we drive both $S_{A}$ and $S_{B}$ toward the transverse direction in the form of a spin-flop transition to reduce the overall Zeeman energy once the induced ferromagnetic moment, defined by $M=S_{A}+S_{B}$, is aligned by the field outward the screen. Consequently, the Néel order defined by $N=S_{A}-S_{B}$ would be pushed toward the longitudinal direction based on the orthogonal geometry with $M$, as shown at the bottom of **Figure S1(b)**. After field application, the two spins switch to the longitudinal axis as shown in **Figure S1(c)**, giving rise to global Néel order forming along the longitudinal direction.

**Supporting information 2∣Polarity check of SHMR examined using W layer**

**
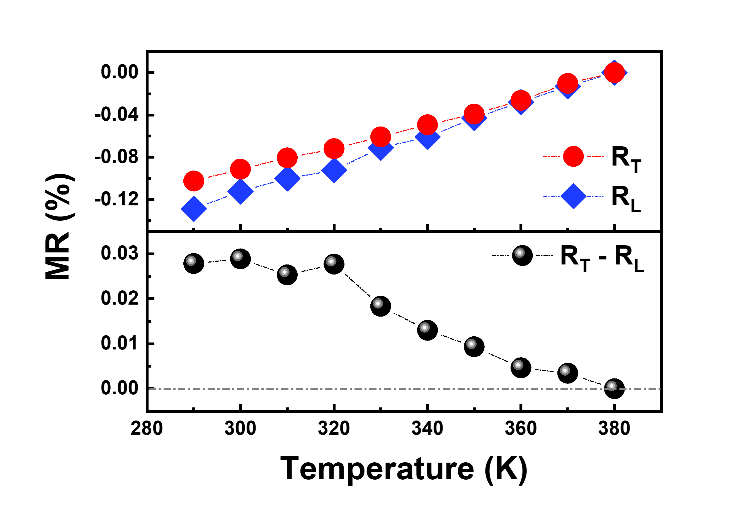
**

**Figure S2.** (Upper panel) Normalized *R_T_* and *R_L_* taken from a NiO/W bilayer, where the *R_380K_* is utilized for the normalization. (Bottom panel) SHMR obtained from the difference between *R_T_* and *R_L._*

As mentioned in the main content of manuscript, the SHMR effect arising from two orthogonal spin-moment configurations in **Figure 1** is independent of spin Hall angle of the heavy metal due to its axis-sensitive nature. Therefore, using heavy metals with opposite spin Hall angle would not change the SHMR because the spin polarization of generated spin current is always in the transverse axis of the device. Only when the Néel order is away from the transverse axis can the SHMR be observed. **Figure S2** exhibits the SHMR of a NiO/W bilayer, in which the positive SHMR also follows the spin-flop mechanism as demonstrated in the main content of manuscript and appears to exhibit a similar temperature-dependent trend as in NiO/Pt. The result suggests that this SHMR-FC methodology is a powerful and broadly applicable platform for AFM-based spintronic technologies.

**Supporting information 3∣Concept of Néel order facilitation in polycrystalline NiO with (111) growth orientation.**


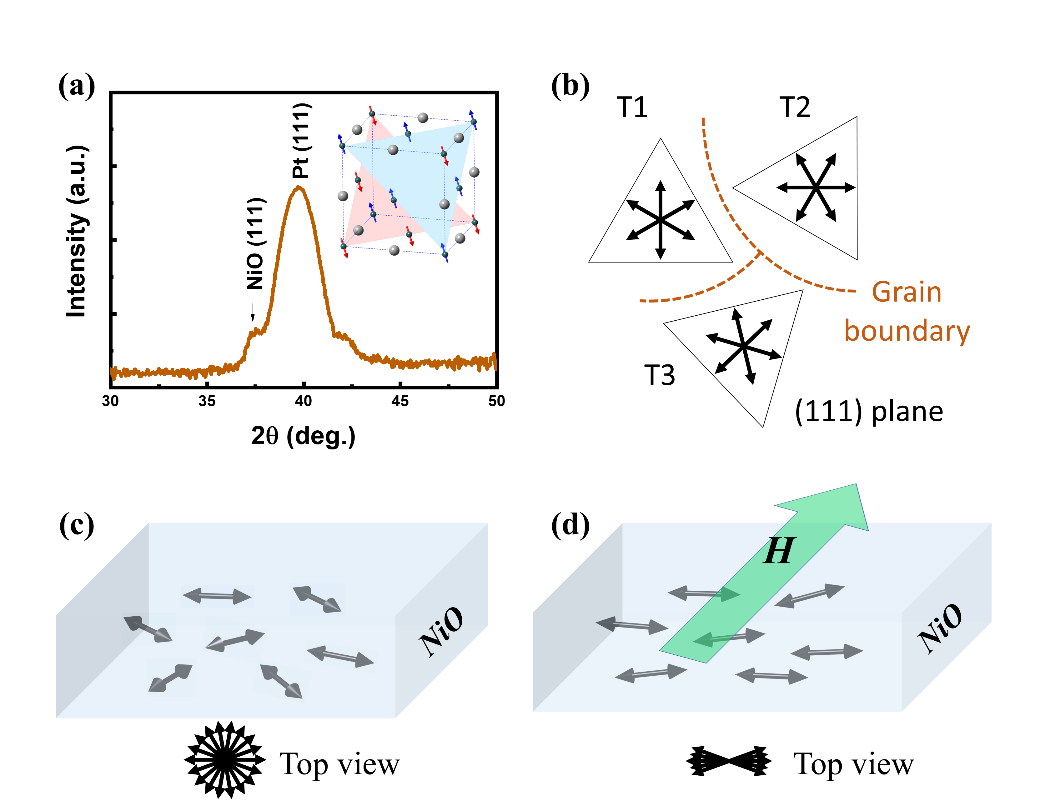


**Figure S3.** (a) X-ray diffraction pattern of the NiO(3)/Pt bilayer with the index of (111) for both NiO and Pt. Inset: G-type spin structure of NiO to show the AFM coupling between two adjacent (111) planes together with the AFM easy-axis in <11$\bar{2}$> family. (b) Possible AFM domain configurations with the <11$\bar{2}$> easy-axis in polycrystalline NiO film, collectively yielding an easy-plane anisotropy. Schematic diagram of the local Néel orders in polycrystalline NiO film (c) before and (d) after an external field is applied. Figures at the bottom of each panel show the corresponding global Néel order in the NiO film, which are isotropic Néel ordering in the plane and anisotropic Néel ordering, respectively.

**Supporting information 4∣Asymmetric SHMR evolution on *R_T_* and *R_L_***


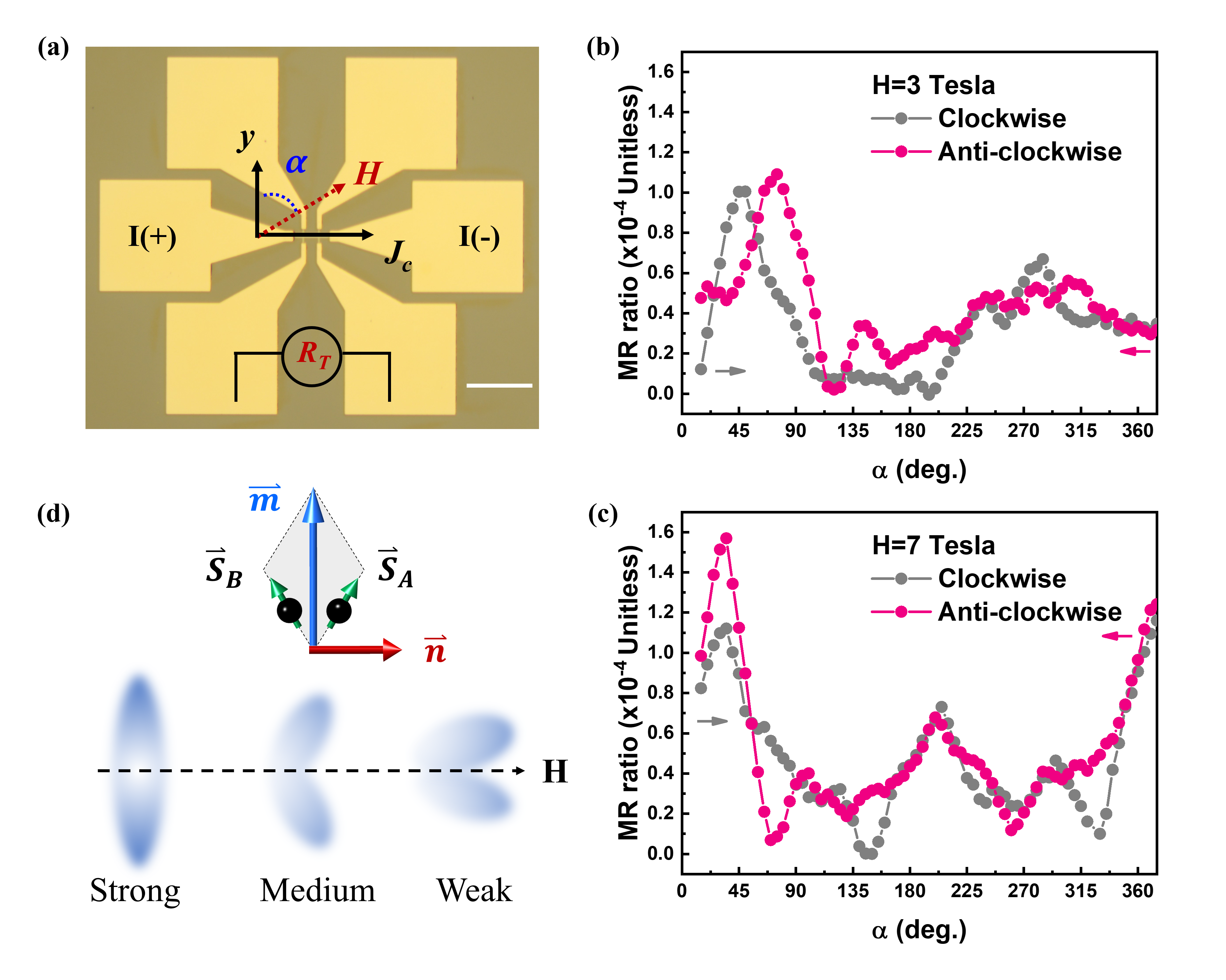


**Figure S4.** (a) Experimental geometry for angle-dependent magnetoresistance (ADMR) measurements of the NiO(1)/Pt bilayer. The angle α is defined between the applied magnetic field and the +y-direction of the device. α is rotated clockwise and counter-clockwise during ADMR to examine the reversibility of the Néel order switching driven by field. (b) ADMR of the NiO(1)/Pt bilayer at H = 3 T and (c) at H = 7 T. (d) Two-spin model describing the magnetic order in NiO, where$S_{A}$ **and** $S_{B}$​ are spins of the two NiO sublattices, $m=S_{A}+S_{B}$​ corresponds to the induced ferromagnetic component that is field-sensitive, and $n=S_{A}-S_{B}$ is Néel order, which is largely field-insensitive. The schematic illustration of the two-spin model for collinear NiO (top) and spin distributions in polycrystalline NiO with strong, medium, and weak AFM exchange stiffness (bottom).

As shown in **Figure S4(b)** and **Figure** **S4(c)**, the ADMR of the NiO(1)/Pt bilayer deviates from the conventional symmetry, where maxima and minima are typically expected at α = 0°/180° and 90°/270°, respectively. This deviation suggests a nontrivial anisotropy energy landscape within the NiO(111) plane, leading to the maxima and minima of the MR to shift with varying α. Increasing the field strength reduces this anomalous anisotropy landscape. Although a 3 T field appears to saturate the SHMR (as shown in **Figure 4(a)** of the main text), it only drives most Néel orders into metastable states. A stronger field of 7 T can overcome the distributed local anisotropy across the NiO(111) plane, restoring a four-fold symmetry in the ADMR. This behavior should result from the weak AFM exchange stiffness case in the two-spin model (**Figure S4(d)**), where spin-flop dynamics dominate. This observation also provides insight into the asymmetric field-dependent MR shown in **Figure 4(a)** of the main text. Specifically, the transverse *R_T_* and longitudinal *R_L_* resistances correspond to α = 0° and α = 90°, respectively. Since these angles do not coincide with the true MR extrema revealed in ADMR, the MR evolution under field naturally appears to be asymmetric.
